# Solvent-Free Determination of Selected Polycyclic Aromatic Hydrocarbons in Plant Material Used for Food Supplements Preparation: Optimization of a Solid Phase Microextraction Method

**DOI:** 10.3390/molecules28165937

**Published:** 2023-08-08

**Authors:** Barbara Benedetti, Marina Di Carro, Chiara Scapuzzi, Emanuele Magi

**Affiliations:** Department of Chemistry and Industrial Chemistry, University of Genoa, 16146 Genoa, Italy; barbara.benedetti@unige.it (B.B.); marina.dicarro@unige.it (M.D.C.); chiara.scapuzzi@edu.unige.it (C.S.)

**Keywords:** polycyclic aromatic hydrocarbons, head space solid phase microextraction, GC-MS, plant contamination, food supplements, circular economy, multivariate optimization, design of experiment

## Abstract

The exploitation of waste and by-products in various applications is becoming a cornerstone of the circular economy. A range of biomasses can be employed to produce food supplements. An example is a particular extract obtained from plant buds (rich in bioactive molecules), which can be easily retrieved from cities’ pruning. In order to safely use this material, its possible contamination by organic pollutants needs to be estimated. A green and simple method to detect priority polycyclic aromatic hydrocarbons (PAHs) in bud samples by head space solid phase microextraction coupled to GC-MS was developed. This strategy, optimized through experimental design and response surface methodology, requires a minimal sample pre-treatment and negligible solvent consumption. The final method was found to be accurate and sensitive for PAHs with mass up to 228 Da. For these analytes, satisfactory figures of merit were achieved, with detection limits in the range 1–4 ng g^−1^, good inter-day precision (relative standard deviation in the range 4–11%), and satisfactory accuracy (88–105%), along with specificity guaranteed by the selected ion monitoring detection. The method was applied to bud samples coming from differently polluted areas, thus helping in estimating the safety of their use for the production of food supplements.

## 1. Introduction

The principles of the circular economy are gaining more and more importance in our society. According to these principles, materials that were historically considered waste have become precious resources. In fact, biomasses derived from a range of human activities and manufacturing can be employed to obtain high-added value products, due to their intrinsic physico-chemical characteristics [1,2]. Diverse plant-derived by-products can be rich sources of bioactive molecules, which can be used, for example, to produce food additives or supplements [3]. Recently, the recycling of cities’ pruning to obtain valuable products has attracted attention [4,5]. Tons of plant branches are removed every year, and may represent more than firewood, especially in the germination period of the plant (late winter/early spring). Indeed, in these weeks, trees are full of buds and young sprouts, namely the young meristematic fresh tissues which later turn into leaves, flowers, or fruits [6]. Buds are characterized by a high content of a range of beneficial phytochemicals, such as polyphenols, thus representing a particularly interesting raw material to obtain food supplements [7]. Their natural scarcity makes the recovery from cities’ pruning a valuable and economically advantageous option. However, city trees are exposed to a certain degree of pollution, also depending on the specific location in the urban area. Therefore, the possible contamination of buds collected from pruning should be estimated, in order to guarantee the safety of the potential food supplements. The anthropic impact is reflected in contamination by organic and inorganic chemicals, and among them, polycyclic aromatic hydrocarbons (PAHs) deserve special attention, since their possible accumulation on urban leaves is widely documented [8,9,10,11,12]. PAHs represent a huge class of substances whose structures are characterized by two or more condensed aromatic rings, which can also present substituents [13]. They are ubiquitously present in the environment, since they are mainly generated by incomplete combustion in a range of natural and anthropic events [14]. The United States Environmental Protection Agency (US EPA) classified sixteen PAHs as priority pollutants, due to their persistence and proven detrimental effects on human health. In particular, seven of them, namely benzo[a]anthracene (BaA), chrysene (CHR), benzo[b]fluoranthene (BbF), benzo[k]fluoranthene (BkF), benzo[a]pyrene (BaPY), indeno[1,2,3-c,d]pyrene (IcdPY), and dibenzo[a,h]anthracene (DahA) are classified as being probably carcinogenic [15]. The presence of PAHs in food supplements has been regulated since 2015 by an EC directive [16], making the quantitation in the final product mandatory. For this purpose, an analytical method specific for these bud extracts was developed in our laboratory (data still not published). However, quality control of the raw material is highly desirable, in order to only use non-contaminated buds to obtain products for human consumption, thus avoiding waste of time and resources.

Among the sample preparation strategies that could possibly be used to determine PAHs in buds, the recent principles of white chemistry suggest the combination of green techniques to achieve reliable instrumental analysis [17]. Microextraction techniques are considered greener than the classical ones, and among these, solid phase microextraction (SPME) is a widespread, simple, and solvent-free technique [18]. It has been applied to the analysis of PAHs in several matrices, both in immersion and head space (HS) modes [19]. The vast majority of the works regard water samples, with a range of diverse coatings tested, such as the conventional polydimethylsiloxane (PDMS) [20,21] or novel materials such as graphitic carbon nitride [22], carbon nanotubes [23], and metal-organic frameworks (MOFs) [24,25]. Regarding plant matrices, HS-SPME has been widely employed for a range of compounds, especially those giving the aromatic profiles [26]. However, few studies have reported the application of this technique to monitor the contamination of plants or trees [27,28]. Indeed, the evaluation of urban plants’ contamination by PAH is reported by more classical analytical approaches, such as solid–liquid extraction, mostly using chlorinated solvents [29,30,31,32].

The present work aimed at the optimization of a simple and green HS-SPME method coupled to analysis by gas chromatography-mass spectrometry for the quantitation of the 16 priority PAHs in *Tilia tomentosa* buds, intended for the production of polyphenol-rich food supplements. The multivariate optimization of the methodology involved two consecutive experimental designs, for a cost- and time-effective development of the method.

The final method was applied to bud samples collected in areas subjected to different anthropic impacts, in order to verify the method’s applicability and detect possible contamination.

## 2. Results and Discussion

The focus of this work was the optimization of the HS-SPME strategy, while a previously optimized GC-MS method was employed for the PAHs detection. Among the wide range of possibilities offered by the SPME coatings, polydimethylsiloxane (PDMS) was selected for this study due to its commercial availability and suitability to extract PAHs, as previously reported [33,34,35]. A preliminary evaluation of an HS-SPME-GC-MS method was performed to set the concentration levels to be spiked onto the sprout pool, in order to have an acceptable sensitivity for the subsequent optimization. The test indicated that a spike of 30 ng g^−1^ was sufficient to detect most of the PAHs’ peaks; thus, this concentration level was kept constant in all the trials of the experimental designs. To select the variables to optimize, both literature information and previous knowledge were considered.

The sample agitation is a significant variable when dealing with HS-SPME of liquid samples, since increasing agitation speed favors the exchange between the liquid and gas phase [18]. Instead, agitation would not seemingly influence the sampling from the solid bud samples and was therefore not considered among the variables of the experimental designs. Furthermore, the desorption temperature (T_DES_) was kept constant at 250 °C. In previous works using similar fiber coatings, a temperature of 270 °C was used for desorption [33,35], but a temperature slightly below this value was chosen to preserve the fiber life, and also according to the manufacturer suggestions. By analyzing blanks after the preliminary experiments, no carry-over was observed, indicating a complete desorption of the considered analytes at the selected temperature. Blank analyses were also performed at the beginning and at the end of every batch of analyses, to ensure the fiber cleanliness.

### 2.1. Optimization of the HS-SPME

#### 2.1.1. First Experimental Design

The response to be maximized was the PAHs’ peak areas obtained from the HS-SPME-GC-MS analysis. To do so, different aliquots (100 mg) of a pool of plant sprouts were spiked to obtain a PAH concentration of 30 ng g^−1^ and subjected to the HS-SPME-GC-MS analyses. A face-centered central composite design was used for both optimization steps, and the following variables were initially taken into account:

- X_1_—Incubation temperature, which corresponded to the exposition temperature (T_EXP_);
- X_2_—Incubation time (t_INC_);
- X_3_—Exposition time (t_EXP_);
- X_4_—Desorption time (t_DES_).

Once all experiments were carried out, the peak areas were used to build the quadratic models (Equation (1) in Section 3) for all analytes. The explained variance (R^2^_ADJ_) of the models and the statistical significance of the model coefficients were evaluated [36]. The PAHs with a molecular mass (MM) greater than that of PYR, namely ranging from 228 to 278 Da (named heavy PAHS, “H-PAHs”), were detected only in a few experiments; thus, the construction of the models was not feasible. The NAPH model was also found to be non-significant. This chemical is ubiquitous and often present in indoor air [37]; thus, accidental contamination may have occurred during this stage, compromising the DoE results. On the other hand, all the other PAHs with a MM up to 202 Da (named light PAHs, “L-PAHs”) showed satisfactory models, whose R^2^_ADJ_ are shown in Table 1, along with the significance of the coefficients.

The T_EXP_ and t_EXP_, as well as their interaction, was found to be significant in influencing the response for most analytes. The model coefficient for both T_EXP_ and t_EXP_ had a positive sign in all cases, meaning that longer expositions at higher temperatures favor the analytes’ sorption onto the PDMS. The importance of these parameters was previously observed in studies regarding PAH quantification by HS-SPME in other matrices [38]. Interestingly, for ACL and AC, t_EXP_ demonstrated a negligible effect, but the interaction between T_EXP_ and t_EXP_ was significant, with a negative sign. At higher T_EXP_ values, the highest peak area was observed at minimum t_EXP_, while the contrary was found at lower T_EXP_ values.

A principal component analysis (PCA) performed on the results [39] showed that the first two principal components (PC) were representative of 85% of the total variance. All analytes with mass from 152 to 252 Da were characterized by a negative and similar loading on PC1, thus indicating a high correlation among the L-PAHs (loading plot reported in Figure S1). Indeed, the conditions providing the best results were rather similar for most of them, consisting of T_EXP_ = 70 °C and t_EXP_ = 50 min. This outcome partially agrees with other works regarding PAH extraction by HS-SPME. For example, Sulej-Suchomska et al. reported 50 °C as the best T_EXP_ value for NAP and FL, and 60 °C as the best value for ANT, FLT, and PY [35].

Even though the mentioned conditions seemed ideal for L-PAHs, the absence of signals of the H-PAHs in almost all the experiments suggested that the selected domain was not suitable for them. Due to these results, a different domain was chosen for the subsequent design, in order to try to improve the signal of all PAHs.

#### 2.1.2. Second Experimental Design

As mentioned, during the first set of experiments, the H-PAHs (specifically BaA, CHR, BbF, BkF, and BaPY) were detected in only two experiments. In these trials, both T_EXP_ and t_EXP_ were at the highest level, thus suggesting the further investigation of only these two variables by modifying the domain toward higher values [21]. Therefore, as indicated in Section 3.3, the range investigated for T_EXP_ was moved from 40–70 °C to 80–100 °C, while t_EXP_ was explored in the range of 60–90 min (prior range 20–50 min). As for the previous design, the areas obtained by the HS-SPME-GC-MS analyses were used to build the quadratic models. The explained variance and coefficient significance of the models obtained are shown in Table 2.

In this new domain, some differences in the effect of the selected variables emerged. For L-PAHs, T_EXP_, always significant in the first domain, showed a lower effect, and was even not significant for some analytes, while the interaction between T_EXP_ and t_EXP_ maintained its significance for most L-PAHs, with similar coefficients compared to those of the first set of models. Regarding H-PAHs, the outcome of this second experimental design confirmed what the first suggested: higher T_EXP_ resulted in an improvement in the signal. In fact, the coefficients of this variable were positive for BaA, CHR, BbF + BkF, and BaPY models. However, even in this modified domain, PAHs with MM above 252 Da (indeno[1,2,3-c,d]pyrene (IcdPY), dibenzo[a,h]anthracene (DahA) and benzo[g,h,i]perylene (BghiPE)) were not detected in all the experiments, thus generating poor models. The positive effect of higher T_EXP_ for the sorption of H-PAHs onto PDMS fibers has been already reported [21,35] and is probably due to their lower vapor pressure, requiring higher temperature to pass to the vapor phase. On the other hand, too-high temperatures generally favor the analytes’ desorption, since the sorption onto the fiber is an exothermic process [40]. This is probably the reason why, for the heaviest PAHs (IcdPY, DahA, and BghiPE), none of the tested conditions produced efficient extraction [41]. Few methods for these PAHs using the same fiber material in HS mode can be found in the literature [21,35] and they generally refer to water samples. It must be noted that the interactions occurring among the analytes and the bud surface may have a strong influence on the passage of the heaviest analytes to the vapor phase.

**Figure 1:**
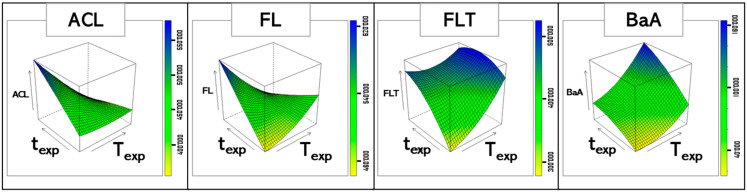
Response surfaces obtained for ACL, FL, FLT, and BaA, indicating the relation among the responses (peak areas) and the investigated variables (t_EXP_ and T_EXP_). A gradual change in the shape of the curve is clearly observed by passing from L-PAHs to H-PAHs.

Figure 1 shows the response surfaces of some of the analytes, which visually highlight their change in shape by increasing the PAH molecular mass; a gradual inversion of the trend with respect to the effect of T_EXP_ on the response can be observed, as already suggested by the values of the models’ coefficients.

Moreover, the strong interaction between T_EXP_ and t_EXP_ for L-PAHs is particularly visible in the FL response surface (Figure 1). Setting T_EXP_ and t_EXP_ at their highest values was necessary to detect H-PAHs, but the maximization of the other signals would have led to the choice of a lower t_EXP_. Therefore, for L-PAHs, the optimal conditions were those identified from the first ED (T_EXP_ = 70 °C, t_EXP_ = 50 min), and guarantee the highest sensitivity. On the other hand, to obtain acceptable sensitivity for H-PAHs, the best conditions were T_EXP_ = 100 °C and t_EXP_ = 90 min. The optimal conditions for the two groups of analytes are shown in Table S1. However, buds spiked at different concentration levels were analyzed by the optimal method for H-PAHs to evaluate the quantitation limits for L-PAHs in “non-optimal” conditions. It must be noted that the optimal method for L-PAHs did not allow the detection of any of the H-PAHs; on the contrary, using the other method, totally acceptable LODs were obtained also for L-PAHs (see Table 3). Hence, the method at higher T_EXP_ and t_EXP_ was chosen as the most suitable for analyzing the real samples. Unfortunately, the analyses of the spiked buds highlighted the presence of a rather intense interferent peak at the retention time of BaPY, thus hindering its determination in the specific sample under study.

### 2.2. Method Performances

Once the optimal HS-SPME conditions were chosen, the overall method was evaluated in terms of linearity range, limit of detection (LOD), limit of quantitation (LOQ), precision, and accuracy. Results are shown in Table 3.

In order to determine the linearity range and build the calibration curves, an estimation of the concentration in real samples was necessary. Therefore, samples collected in urban areas characterized by different pollution levels (low, medium, and high) were spiked with 100 ng g^−1^ of PAHs and 10 ng g^−1^ of IS and analyzed by the optimal method for H-PAHs. The PAHs level in each sample was roughly estimated by comparing the non-spiked samples with the spiked ones to identify the concentration range for the calibration.

LODs and LOQs in matrix were determined by considering the noise signal in the chromatogram of the non-spiked low-polluted sample. When the sample was found to be contaminated, the noise in a region as close as possible to the peak was taken into account. LODs and LOQs were the concentrations providing signal-to-noise (S/N) ratio values of 3 and 10, respectively, and are summarized in Table 3. Then, in-matrix calibration curves were built by adding increasing concentrations of PAHs to the low-pollution sample, setting the following levels: LOQ level, 10, 50, 100, and 200 ng g^−1^, with a constant IS concentration of 10 ng g^−1^. Quantitation was performed by following the internal standard strategy; thus, the ratios among the areas of each PAH and the corresponding deuterated internal standard (Table S2) were used to build the calibration curves. The linear dynamic range was approximately two orders of magnitude for L-PAHs, from 3 or 4 ng g^−1^ to 200 ng g^−1^, while it was smaller for H-PAHs, due to higher LOQ levels. These analytes also generally suffer from lower sensitivity using classical liquid-injection mode in GC analysis [39].

Method precision was assessed by preparing and analyzing six sample aliquots spiked at the same concentration (100 ng g^−1^). These analyses were performed during different days; thus, the obtained results can be considered as the intermediate precision. The relative standard deviation (RSD) was satisfactory, except for BaA and BbF + BkF, for which values of 30–40% were observed. The best performances were instead reached for L-PAHs, for which RSD was in the range 3.4–11.2%. The lower precision for the H-PAHs may be ascribed to the observed broader peaks, which are more influenced by the noise signal, as well as the worse fit of the calibration curve. Moving to higher concentrations could probably lead to better precision, but this was not necessary in our study due to the rather low amounts detected in the samples (see Section 2.3). Trueness was assessed by considering the six samples spiked at 100 ng g^−1^, but was not used to build the calibration curves. Values from 88% to 105% were obtained, indicating rather satisfactory accuracy. The only exceptions were again BaA and BbF + BkF, for the same reasons already discussed. The non-ideal performance of the method for the heaviest analytes may suggest its use as a screening tool for them.

The method was compared with more traditional ones regarding the PAHs’ extraction from comparable plant material, such as leaves and pine needles (see Table S3). The sensitivity of the proposed method is comparable with the values reported by De Nicola et al. [12], but lower than those reported in other works in which PAHs were determined in leaves and pine needles by solid–liquid extraction [9,42] or by matrix solid phase dispersion [43]. In these papers, nevertheless, the sample weight was 5 or 10 g and the volume of solvent employed for the extraction ranged from 40 to 100 mL, except for the case of De Nicola (2016) [43], in which 0.25 g and 20 mL were used. These sample amounts are rather higher than that used in the present paper (0.1 g), while solvent volumes are not even comparable (being virtually 0 in the proposed strategy). Regarding trueness, values obtained are in some cases better than those reported in the considered papers, while precision is comparable or slightly higher.

**Figure 2:**
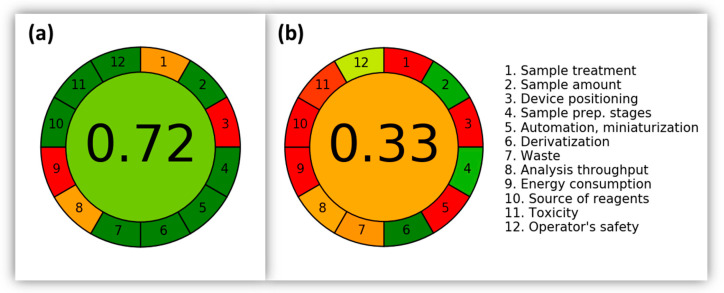
Pictograms obtained by using the AGREE metric tool for the assessment of method greenness: comparison of (**a**) the present method and (**b**) a recent method for the determination of PAHs in plant material (oak leaves and pine needles) [43].

For a more complete evaluation, a comparison in terms of greenness was performed between the developed method and one of the cited works, by considering the more recent study [43], which is characterized by the lowest amounts of the sample and the volume of solvent used. The AGREE system [44] was used as a greenness metric tool to compare the two methods, resulting in a definitely higher score of the proposed method compared to the other approach (see Figure 2). Indeed, 0.72/1 was gained (0.33/1 for the other work), indicating a clear improvement in the strategy in terms of eco-friendliness. The use of high volumes of toxic solvents and a higher number of preparation steps dramatically influenced the score for the other method.

### 2.3. Application to Real Samples

**Figure 3:**
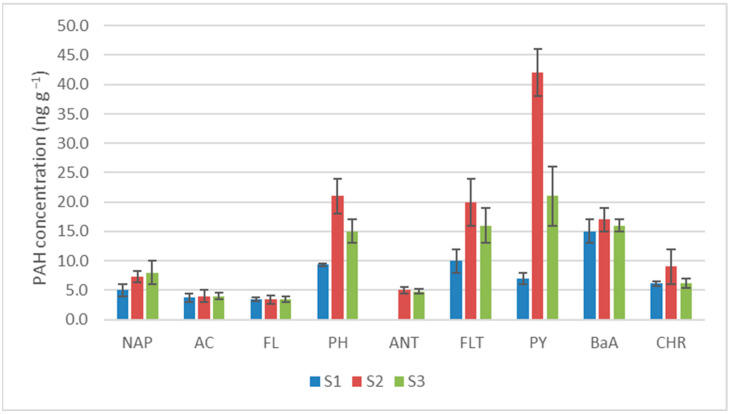
Quantitation of PAHs in the three bud samples collected in different areas of the city of Turin (Italy).

*Tilia tomentosa* bud samples were used to verify the applicability of the method and to estimate the concentration of PAHs in buds coming from areas subjected to different degrees of anthropic impact. Indeed, buds were collected in three areas of the Italian city of Turin, characterized by low, medium, and high pollution, mainly given by vehicular traffic. An example chromatogram is shown in Figure S2 (Supplementary Information), highlighting the most concentrated PAHs found in the samples. The quantitation results, shown in Figure 3 (Table S4 for detailed values), indicate the presence of most of the analytes, but at very low concentration levels. All analyzed samples showed contamination levels in the range of 3.4–42 ng g^−1^, with the highest concentrations found for PH, FLT, PY, and BaA. AC and FL were rather near to their LOQ levels, namely below 5 ng g^−1^, independently from the area of the buds’ collection. In all other cases, the low-pollution sample was characterized by the lowest concentration, in line with what was expected. On the other hand, no significant trend was observed by passing from the medium-polluted to the highly polluted sample. The distance between the two collection sites was actually not huge and the mobility of the contaminants through particulate matter is highly probable [45,46]. The preponderance of PH, FLT, and PY was observed in the past in the same Italian region, when a monitoring of PAH contamination highlighted that the mentioned analytes and CHR accounted for more than 80% of the total PAHs [47].

Unfortunately, no information could be retrieved regardisng BaPY, IcdPY, DahA, and BghiPE, for which the developed method was not suitable (for the different reasons already discussed). The critical analytes for which the EC regulation established concentration limits in food supplement are BaPY, BaA, CHR, and BbF; even though BaPY was not quantifiable, the determination of the other priority PAHs is still of great importance within quality control activities. The highest concentrations were found for PH, FLT, PY, and BaA, but the first four compounds are not subjected to any legal limit in food supplements. These results suggested that the collected samples were characterized by a general low contamination. Nevertheless, it is of utmost importance to check for the presence of BaPY in the final extracts, since its quantification in *Tilia tomentosa* buds was not feasible with the proposed technique.

## 3. Materials and Methods

### 3.1. Chemicals and Reagents

A stock standard solution of the 16 priority PAHs, at a concentration of 2000 µg mL^−1^ each, in acetone/benzene, was purchased from Dr. Ehrenstorfer GmbH (Augsburg, Germany). The contained analytes are the following: naphthalene (NAP), acenaphthylene (ACL), acenaphthene (AC), fluorene (FL), phenanthrene (PH), anthracene (ANT), fluoranthene (FLT), pyrene (PY), benzo[a]anthracene (BaA), chrysene (CHR), benzo[b]fluoranthene (BbF), benzo[k]fluoranthene (BkF), benzo[a]pyrene (BaPY), indeno[1,2,3-c,d]pyrene (IcdPY), dibenzo[a,h]anthracene (DahA) and benzo[g,h,i]perylene (BghiPE). A standard solution containing 4 completely deuterated PAHs in toluene at a concentration of 1000 µg mL^−1^ each was purchased from Dr. Ehrenstorfer GmbH (Augsburg, Germany). The deuterated compounds were d- naphthalene (d-NAP), d-acenaphthene (d-AC), d-phenanthrene (d-PH) and d-perylene (d-PE). Chromatographic grade acetone, used to dilute the stock standard solutions, was from VWR Chemicals (Fontenay-sous-Bois, France).

### 3.2. Instrumentation and GC-MS Analysis

The instrumentation used for the HS-SPME-GC-MS analysis was a 7890 A gas chromatograph coupled to a 5975 C MSD mass spectrometer, both from Agilent (Agilent Technologies, Santa Clara, CA, USA). The GC-MS was equipped with a Gerstel MultiPurpose Sampler (MPS, by Gerstel GmbH & Co., KG, Mülheim an der Ruhr, Germany). This system was exploited to perform automated HS-SPME, thanks to the support for the SPME syringe. A polydimethylsiloxane (PDMS)-coated fiber with a thickness of 100 μm from Supelco (Bellefonte, PA, USA) was employed throughout the work.

The same GC-MS method was used independently from the prior HS-SPME treatment. The injector where the fiber was thermally desorbed was set at 250 °C. A 36 min chromatographic run permitted PAH separation on a Rxi^®^-5Sil MS capillary column (30 m × 0.25 mm ID, 0.25 µm film thickness) from Restek Italy (Cernusco sul Naviglio, Italy). Helium was used as carrier gas at a flow rate of 1.2 mL min^−1^ and the following oven temperature program was employed: initial temperature 60 °C, (kept constant for 2 min); first ramp to 280 °C at a rate of 10 °C min^−1^ (kept constant for 5 min); second ramp to 300 °C at 10 °C min^−1^, held for 10 min. Positive electronic ionization (EI+) was employed at the GC-MS interface with an electron energy of 70 eV. The transfer line and ion source temperatures were 280 °C and 230 °C, respectively. Mass spectrometry detection was performed by a single quadrupole operating in single ion monitoring (SIM) mode; for each PAH, the *m*/*z* value corresponding to the molecular ion (M+) was used to optimize sensitivity. The monitored *m*/*z* values for both standards and internal standards are shown in Table S2 (Supplementary Information). As frequently reported in the literature, benzo[b]fluoranthene and benzo[k]fluoranthene were partially co-eluted; thus, they were quantified as the sum. Data acquisition and processing were achieved by using the Agilent GC/MSD MassHunter Acquisition Software (version B.07.00) and the MassHunter MSD Data Analysis software (version B.04.01).

### 3.3. Experimental Designs

Two sequential experimental designs were performed for the procedure optimization. The response to be maximized was the PAHs’ peak areas obtained from the HS-SPME-GC-MS analysis. To do so, different aliquots of a pool of plants sprouts were spiked with a known amount of PAHs (3 ng) and subjected to the HS-SPME-GC-MS analysis. A face-centered central composite design was used for both optimization steps and the following variables were initially taken into account:

- X_1_—Incubation temperature, which corresponded to the exposition temperature (T_EXP_);
- X_2_—Incubation time (t_INC_);
- X_3_—Exposition time (t_EXP_);
- X_4_—Desorption time (t_DES_).

In the face-centered design, the α value is set as equal to 1 [48]. Thus, three coded levels were selected for each variable, namely −1, 0, and +1, which corresponded to the real values indicated in Table 4.

Three replicates of the center point were added, leading to a total of 27 experiments. The whole set of performed experiments is reported in Table S5. This design allowed the computation of the quadratic mathematical model:

(1) $$Y=b_{0}+\overset{k}{\underset{i=1}{\sum }}b_{i}x_{i}+\overset{k}{\underset{1\leq i<j}{\sum }}b_{ij}x_{i}x_{j}+\overset{k}{\underset{i=1}{\sum }}b_{ii}x_{i}{}^{2}$$

where *Y* is the response (PAH peak area), *k* is the number of the variables and *b_i_*, *b_ii_*, and *b_ij_* are the coefficients of the linear terms, of the quadratic terms, and of the interactions, respectively.

The same type of experimental design was used in the second step of the optimization, but investigating only the variables that were found to be significant after the first experimental design, namely T_EXP_ and t_EXP_. Furthermore, a different domain was chosen, based on the outcomes of the previous tests, with new levels shown in Table 5.

The 11 performed experiments are reported in Table S6. The same responses as before were considered and quadratic mathematical models were built to identify the optimum.

The open-source software CAT (Chemometric Agile Tool, version 2019) was employed for the computation of the response surface models.

### 3.4. Bud Samples

*Tilia tomentosa* Moench fresh buds were collected during the spring of 2021, in 3 urban and sub-urban areas of the city of Turin (Italy), with different degrees of environmental pollution. Specifically, sample S1 was from the Superga Hill (low pollution), sample S2 was from Ignazio Michelotti Park (medium pollution), and sample S3 was from Massimo D’Azeglio Avenue (high pollution). Each sample was constituted by a pool of buds belonging to different plants, but collected in the same area.

Whole buds were stored at −18 °C until treatment. Approximately three grams of material were thawed and minced through a small mixer, and 0.1 g aliquots were accurately weighed. Two aliquots were weighed for each sample, fortified with IS (IS concentration equal to 10 ng g^−1^), and left under a fume hood overnight to let the solvent evaporate. The following day, they were subjected to the HS-SPME-GC-MS analysis.

### 3.5. Optimal HS-SPME Method

The optimal method for H-PAHs with MM 202–252 Da was found to also be completely acceptable for the analysis of the lighter PAHs (see Section 2.2). The conditions set for this method were as follows: sample incubation for 20 min at 100 °C; fiber exposition for 90 min at the same temperature; and final desorption for 3 min at 250 °C in the GC injector.

## 4. Conclusions

The developed method demonstrated rather good analytical performances for the aim of detecting PAH contamination in bud samples. This is of utmost importance for the consequent use of this material for food supplements. Most of the literature studies regarding PAH determination in urban plant leaves basically use chlorinated solvent extraction. On the contrary, the proposed HS-SPME-GC-MS methodology is almost solvent-free, since it only requires a few µL of acetone to add internal standards, thus producing negligible waste. The AGREE metric confirmed the greenness of the method, by assigning a 0.72 score (out of 1, considered as the ideal score). The exploitation of the experimental design allowed the rational optimization of the methodology and highlighted its limits regarding the heaviest PAHs.

All analyzed samples coming from cities’ pruning showed contamination levels in the range of 3.4–42 ng g^−1^. However, the PAHs with the highest MM could not be determined due to method unsuitability as well as the presence of interference species. It is therefore mandatory to verify the presence of this class of contaminants directly in the bud organic extract used for the food supplements’ production. However, the proposed strategy for the raw material is simple, cheap, and eco-friendly, thus representing an advantageous screening tool to guarantee the quality of the final product.

## Figures and Tables

**Table 1:** Explained variance and significant coefficients of the models obtained for L-PAHs (first DoE).

PAHs	R^2^_ADJ_	Significant Coefficients
Acenaphthylene (ACL)	63.06	T_EXP_, T_EXP_^2^, T_EXP_ × t_EXP_
Acenaphthene (AC)	74.85	T_EXP_, T_EXP_^2^, T_EXP_ × t_EXP_
Fluorene (FL)	90.34	T_EXP_, t_EXP_, T_EXP_ × t_EXP_
Phenanthrene (PH)	96.21	T_EXP_, t_EXP_, T_EXP_^2^, T_EXP_ × t_EXP_
Anthracene (ANT)	95.41	T_EXP_, t_EXP_, T_EXP_^2^, T_EXP_ × t_EXP_
Fluoranthene (FLT)	77.71	T_EXP_, t_EXP_, T_EXP_ × t_EXP_
Pyrene (PY)	70.05	T_EXP_, t_EXP_, T_EXP_ × t_EXP_

**Table 2:** Explained variance and significant coefficients of the models obtained by performing the second DoE.

PAHs	R^2^_ADJ_	Significant Coefficients
NAP	65.78	t_EXP_
ACL	85.03	T_EXP_, t_EXP_, T_EXP_ × t_EXP_
AC	75.03	T_EXP_, T_EXP_ × t_EXP_
FL	75.01	T_EXP_ × t_EXP_
PH	92.98	t_EXP_, T_EXP_^2^, T_EXP_ × t_EXP_
ANT	90.16	t_EXP_, T_EXP_ × t_EXP_
FLT	85.45	T_EXP_, t_EXP_, T_EXP_ × t_EXP_
PY	70.52	t_EXP_
BaA	85.85	T_EXP_, t_EXP_
CHR	68.87	T_EXP_, t_EXP_
BbF + BkF *	60.63	T_EXP_, t_EXP_
BaPY	74.44	T_EXP_, t_EXP_

* Partially co-eluted peaks, thus considered as the sum.

**Table 3:** Figures of merit of the overall HS-SPME-GC-MS method (employing the HS-SPME optimal conditions for H-PAHs).

Analyte	Linear Range (ng g^−1^)	R^2^	LOD (ng g^−1^)	LOQ (ng g^−1^)	Inter-Day Precision (*n* = 6)	Trueness (*n* = 6)
NAP	LOQ-200	0.9967	1.4	4.6	9.1%	88 ± 8
ACL	LOQ-200	0.9967	1.3	4.3	4.9%	98 ± 5
AC	LOQ-200	0.9966	1.1	3.6	4.5%	98 ± 4
FL	LOQ-200	0.9970	1.2	4.0	3.5%	101 ± 3
PH	LOQ-200	0.9951	0.9	3.1	3.4%	95 ± 3
ANT	LOQ-200	0.9954	1.3	4.3	7.3%	105 ± 8
FLT	LOQ-200	0.9912	1.4	4.8	7.9%	95 ± 8
PY	LOQ-200	0.9945	1.2	3.9	11.2%	95 ± 11
BaA	LOQ-200	0.9697	3.0	10.0	34.7% *	*130 ± 45*
CHR	LOQ-200	0.9782	1.7	5.8	20.9%	103 ± 22
BbF + BkF	LOQ-200	0.9167	4.4	14.7	*43.6%*	*137 ± 60*

* values in italics indicate non satisfactory results. The method is considered as a screening tool for BaA, BbF and BkF, since lower accuracy and precision were observed.

**Table 4:** Coded levels and real values of the variables studied in the first experimental design.

Coded Values	Real Values			
	X_1_	X_2_	X_3_	X_4_
	T_EXP_ (°C)	t_INC_ (min)	t_EXP_ (min)	t_DES_ (min)
−1	40	20	20	1
0	55	35	35	3
+1	70	50	50	5

**Table 5:** Coded levels and real values of the variables studied in the second experimental design.

Coded Values	Real Values	
	X_1_	X_3_
	T_EXP_ (°C)	t_EXP_ (min)
−1	80	60
0	90	75
+1	100	90

## Data Availability

The data presented in this study are available on request from the corresponding author.

## Supplementary Materials

The following supporting information can be downloaded at: https://www.mdpi.com/article/10.3390/molecules28165937/s1, Supplementary Information (including Figures S1–S2 and Tables S1–S6).
