# Supplementary material for: Solvent-Free Determination of Selected Polycyclic Aromatic Hydrocarbons in Plant Material Used for Food Supplements Preparation: Optimization of a Solid Phase Microextraction Method
Source: Molecules. 2023 Aug 8;28(16):5937. doi: 10.3390/molecules28165937 (PMC10459292; doi:10.3390/molecules28165937)
Supplement: Supplementary file 1 [file molecules-28-05937-s001.zip › molecules-2508729-supplementary.pdf]

Supplementary Information

Solvent-free determination of selected polycyclic aromatic hydrocarbons in plant material used for food supplements preparation: optimization of a solid phase microextraction method

Barbara Benedetti, Marina Di Carro, Chiara Scapuzzi and Emanuele Magi

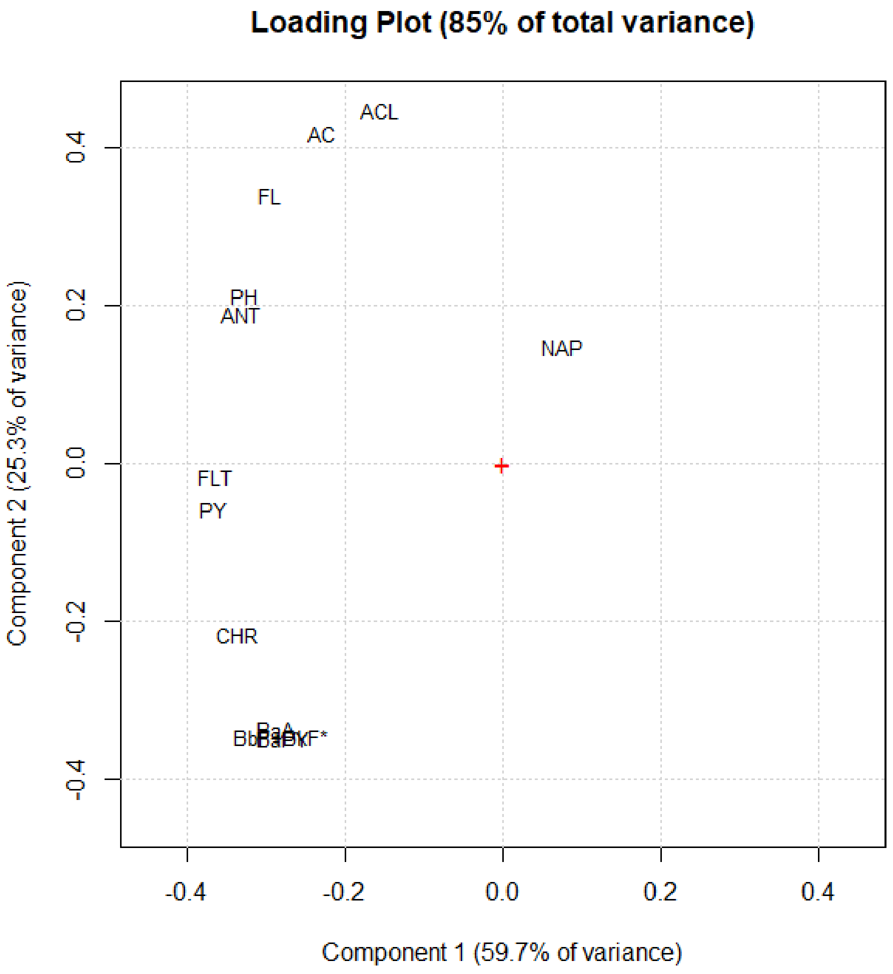

Figure S1. Loading plot of the PCA, performed on the areas obtained by the experiments of the first experimental design. The similar loadings on PC1 indicate a correlation among most analytes.

Table S1. Optimal values of the studied variables to obtain maximum sensitivity for light and heavy PAHs (MM: 128-202 Da and 228-278 Da, respectively).

**Variable**	**Optimal value for L-PAHs**	**Optimal value for H-PAHs**
t_{EXP}	50 min	90 min
T_{EXP}	70 °C	100 °C
t_{INC}	20 min	20 min
t_{DES}	3 min	3 min

Table S2. Selected ions for SIM detection and retention times of the 16 PAHs under study as well as the internal standards.

Compound	Selected ion in SIM mode (m/z)	Retention time (min)	Assigned internal standard
	Analytes		
Napthatlene (NAP)	128	9.11	d-NAP
acenaphthylene (ACL)	152	12.84	d-AC
acenaphthene (AC)	154	13.26	d-AC
fluorene (FL)	166	14.50	d-PH
phenanthrene (PH)	178	16.76	d-PH
anthracene (ANT)	178	16.89	d-PH
fluoranthene (FLT)	202	19.60	d-PH
pyrene (PY)	202	20.12	d-PH
benzo[a]anthracene (BaA)	228	23.00	d-PH
chrysene (CHR)	228	23.08	d-PH
benzo[b]fluoranthene (BbF)*	252	25.66	d-PH

benzo[k]fluoranthene (BkF)*	252	25.74	d-PH
benzo[a]pyrene (BaPY)	252	26.55	d-PH
indeno[1,2,3-c,d]pyrene (IcdPY)	276	30.58	d-PE
dibenzo[a,h]anthracene (DahA)	278	30.72	d-PE
benzo[g,h,i]perylene (BghiPE)	276	31.37	d-PE
Internal standards			
d-Napthatlene (d-NAP)	136	9.06	
d-acenaphthene (d-AC)	164	13.19	
d-phenanthrene (d-PH)	188	16.71	
d-perylene (d-PE)	264	26.74	

* Benzo[b]fluoranthene and benzo[k]fluoranthene are considered as a unique peak (partially coeluted).

Table S3. Comparison of the present method with others in the literature, in terms of the main figures of merit and method characteristics.

Reference	**LOQ** (ng g^{-1})	**Precision** (RSD)	**Trueness**	**Sample amount** (g)	**Sample preparation**
Present work	3.1-14.7	3.4-20.9 %	88-105 %	0.1	SPME
Orecchio et al. (Ref 9)	0.2-0.5	10-20 %	76-102 %	5	SLE[a]
Ratola et al. (Ref 42)	0.7-2.3	5.8-8.2 %	72-100 %	10	SLE
De Nicola et al. (Ref 12)	2.6-8	4-15 %	≈ 70%	5	SLE
De Nicola et al. (Ref 43)	0.1-3.4	2.4-12 %	85-143 %	0.25	MSPD[b]

a- Solid-Liquid Extraction
b- Matrix Solid Phase Dispersion

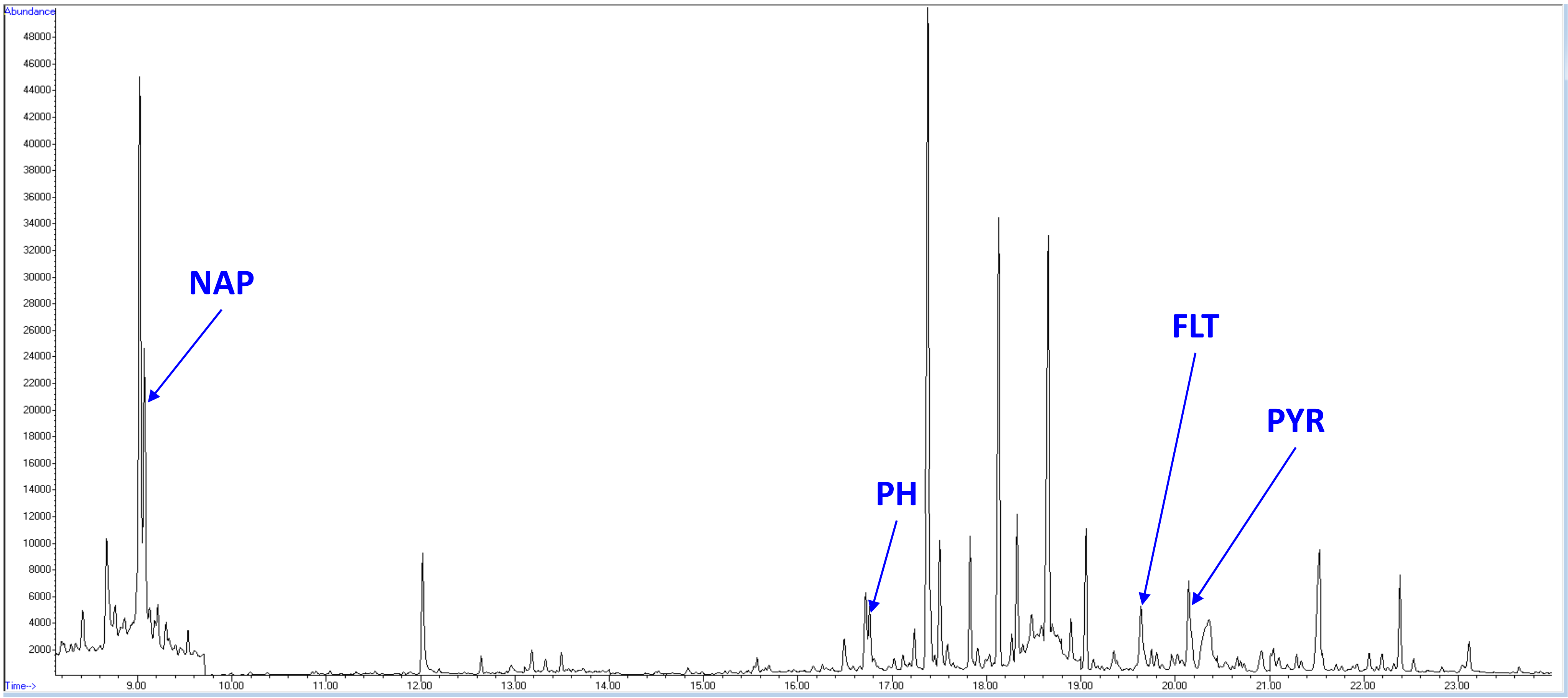

Figure S2: GC-MS chromatogram in Total Ion Current (TIC) of sample S3. The most concentrated analytes are indicated.

Table S4. Results of the quantitative analysis, expressed as ng g^{-1} of fresh buds' samples ± standard deviation (SD) (n=4).

Analyte	Sample S1	Sample S2	Sample S3
		ng g^{-1} ± SD	
NAP	5 ± 1	7.3 ± 0.9	8 ± 2
ACL	< LOD	< LOD	< LOD
AC	3.7 ± 0.7	4 ± 1	4.0 ± 0.5
FL	*3.4 ± 0.3 **	*3.4 ± 0.7 **	*3.5 ± 0.5 **
PH	9.3 ± 0.2	21 ± 3	15 ± 2
ANT	< LOD	5 ± 0.6	4.8 ± 0.4
FLT	10 ± 2	20 ± 4	16 ± 3
PY	7 ± 1	42 ± 4	21 ± 5
BaA	15 ± 2	17 ± 2	16 ± 1
CHR	6.1 ± 0.4	9 ± 3	6.1 ± 0.8
BbF+ BkF	< LOD	< LOD	< LOD

*Values at the LOQ level

Table S5. Experiments performed within the first experimental design; real values of each variable are reported.

Experiment	X_1<br>T_{EXP} (°C)	X_2<br>t_{INC} (min)	X_3<br>t_{EXP} (min)	X_4<br>t_{DES} (min)
1	40	20	20	1
2	70	20	20	1
3	40	50	20	1
4	70	50	20	1
5	40	20	50	1
6	70	20	50	1
7	40	50	50	1
8	70	50	50	1
9	40	20	20	5
10	70	20	20	5
11	40	50	20	5
12	70	50	20	5
13	40	20	50	5
14	70	20	50	5
15	40	50	50	5
16	70	50	50	5
17	40	35	35	3
18	70	35	35	3

19	55	20	35	3
20	55	50	35	3
21	55	35	20	3
22	55	35	50	3
23	55	35	35	1
24	55	35	35	5
25	55	35	35	3
26	55	35	35	3
27	55	35	35	3

Table S6. Experiments performed within the second experimental design; real values of each variable are reported.

Experiment	**X_1**	**X_3**
	T_{EXP} (°C)	t_{EXP} (min)
1	80	60
2	100	60
3	80	90
4	100	90
5	80	75
6	100	75
7	90	60
8	90	90
9	90	75
10	90	75
11	90	75
